# Values Scale for Positive Youth Development in Sport

**DOI:** 10.3390/ejihpe14100175

**Published:** 2024-09-25

**Authors:** Rosendo Berengüí, Francisco J. Parra-Plaza, María Á. Castejón

**Affiliations:** Faculty of Education, Universidad Católica de Murcia (UCAM), 30107 Murcia, Spain

**Keywords:** values, moral development, positive youth development, youth sport, psychometric properties

## Abstract

Adolescence is a period of special significance for the development of personal and social values. Positive adolescent development (PYD) can be an ideal perspective from which to promote values in young people through their sports practice. The aim of the study was to adapt and analyse the measurement properties of the Values Scale for Positive Youth Development for use in the context of sport in young athletes. A total of 599 adolescents, competitors of different sports modalities, participated in the study. Confirmatory factor analysis and reliability and invariance analyses were performed. The results confirmed a good model fit, with adjustment indexes (CFI, GFI and AGFI) above 0.90 and error (RMSEA and SRMR) below 0.08. The factor loadings above 0.50 were obtained for all items. Adequate reliability of the scales was also confirmed, between 0.72 and 0.89, and gender, age and sport invariance were confirmed. In conclusion, the analysed scale is a valid and reliable instrument with adequate psychometric properties, which makes it an appropriate assessment tool to be used in sports contexts, based on the positive adolescent development approach.

## 1. Introduction

Adolescence is an important period, between the ages of 10 and 19 [1,2], in the development of autonomy and identity [3], in which there is a progressive move from external (parental) regulation to self-regulation, with young people becoming increasingly responsible for their own decisions about values and their own behaviour through a process of internalisation of moral values [4]. It is also a period of rapid development that offers an opportunity for the promotion of values that may be more durable and enduring [5]. Several studies have shown that values develop throughout childhood and adolescence, and furthermore that the likelihood of value change decreases with age [6,7]. Therefore, these appear to be critical age periods in the development of values, and a comprehensive understanding of values during adolescence and young people’s experiences with values in different contexts is currently lacking [8].

Along with their family and the educational centre, sport is one of the most important contexts in the process of a child’s formation and development. Participation in sports activities has many benefits, such as improved physical health and decreased symptoms of depression or anxiety [9,10], and is related to a wide range of positive personality traits [11]. Additionally, physical activity and sport programmes in school and community contexts have the potential to foster positive development in relation to social and emotional learning [12]. However, despite evidence of the potential benefits of sports participation, mere participation is not considered sufficient to obtain them [13]. Sport can become an extraordinary context for transmitting a wide range of important personal and social values to children and adolescents, provided that there is an appropriate organisation and educational focus [14,15,16,17] that intentionally incorporate best practices to promote learning and life skill development through sport [18].

One perspective of growing relevance in recent times in the fields of development, psychology and sport is the positive youth development (PYD) approach. It is a strengths-based perspective, focused on harnessing the abilities and potential that come naturally to adolescents to achieve healthy development and adaptive functioning [19,20], and at the same time, the capacity of young people to mitigate their risk and problem behaviours [21]. Although the term PYD has been used in different ways and in different contexts, there are some similarities between the different models, such as the emphasis on the strengths of young people, the plasticity of their development, internal developmental assets such as psychosocial competence and external developmental assets such as community influence [22]. All of this brings the strengths of the individual and the resources available in the environment (positive adult relationships or mentoring opportunities) into mutually beneficial interaction [19,23,24].

At a global level, the PYD approach focusses on four essential domains [25,26]: assets (young people are able to achieve desirable outcomes as they have the necessary resources, skills and competencies), agency (they can influence and make their own decisions about their lives, set their own goals and act on their own decisions), contribution (they can engage in positive developmental change for themselves and their communities) and an enabling environment (the environment can maximise their assets, their capacity to act, their access to services and opportunities, while enhancing their ability to avoid risks and protect themselves).

In sport, the PYD approach seeks to examine the ways in which sport and physical activity can be used to optimise young people’s internal strengths and foster positive sport experiences [27]. Different conceptual frameworks emphasise attention to the relationships between the individual and others (such as teammates, coaches and parents) and to the sport context, such as the organisational structure of the sport club or the characteristics of the community in which the young person is involved [28]. In addition, the PYD-based programmes developed have demonstrated a positive and significant impact on the development of social skills, social competence, positive relationships, social support or life skills, among other benefits [20,29,30,31]. Therefore, PYD seems to be an ideal perspective from which to promote values in young people through its practice.

For the assessment of values and the intervention programmes carried out for their development, classic instruments have usually been used, such as, for example, the Rokeach-Value Survey [32], the Schwartz-Value Survey [33,34] or the Personal Values Questionnaire [35]. Furthermore, there are instruments for the assessment of values in the sport context, such as the Youth Sport Values Questionnaire (YSVQ) [36,37], the Spirit of Sport Values scale [38] or the Sport and Olympic Values Questionnaire [39]. However, there is a gap in terms of instruments based on the PYD approach.

A scale that can be considered of interest in this context is the Values Scale for Positive Youth Development [40]. The authors set out to construct a scale with adolescent values as a construct to be studied within the framework of the PYD. The scale was designed for use in school contexts, being in support of the school having a primary role in the moral and socio-emotional education of students, with a view to promoting competences that will enable young people to make their contribution to society and successfully face their personal and professional lives [40,41].

The authors carried out a review of the various traditional models and instruments for the assessment of values, such as those mentioned above [33,34,35], in addition to the Values Profile [42], the Goals Questionnaire for Adolescents [43] and the Socio-personal Values Questionnaire for Coexistence [44]. Although most of these instruments were supported by a solid theoretical basis and their use was consolidated within values research, all of them were far from the theoretical approach of assessing values related to the promotion of PYD. Different problems were also encountered, such as being oriented to the study of cultural differences in values, being focused on other specific areas unrelated to PYD, or not having appropriate psychometric properties [40].

Its proposal, the approach adopted and the appropriate psychometric properties demonstrated by the instrument make its adaptation to sport possible, interesting and of relevant value. Therefore, the aim of this study was to adapt and analyse the measurement properties of the Values Scale for Positive Youth Development in Sport for use in the context of sport in young athletes.

## 2. Materials and Methods

### 2.1. Participants

The sample consisted of a total of 599 adolescents (392 boys and 207 girls) aged 11 to 19 years (*M* = 14.74, *SD* = 2.40) who were members of different sports clubs in the regions of Alicante, Murcia and Granada (Spain). Athletes competed in organised and official leagues of soccer (42.09%), futsal (18.21%), basketball (29.73%) and handball (9.97%). A total of 50 clubs participated with teams in different categories of competition, from Under-12 (11–12 years) to Under-19 (17–19 years): 19 clubs of soccer, 10 of futsal, 14 of basketball and 7 of handball.

The mean age of starting in the sport was 6.83 years (*SD* = 2.21), and a mean experience of 5.27 years (*SD* = 3.11) competing in their sport. The average number of training days per week was 2.95 (*SD* = 0.88) and 4.91 h (*SD* = 1.57) of training per week.

### 2.2. Measures

The Values Scale for Positive Youth Development is a 24-item scale developed by Antolín et al. [40] that assesses the importance that adolescents attach to a set of values of particular relevance in PYD.

The study of the scale confirmed evidence of validity focused on the dimensionality of the instrument, with a structure of eight first-order factors and three second-order factors. The first-order factors are: Pro-Sociality (importance given to actions of help, collaboration and care for others), Social Commitment (relevance of active participation in the community), Justice and Equality (interest in achieving a socially just and equal world), Responsibility (importance given to personal responsibility and taking responsibility for one’s own actions), Integrity (importance given to acting on the basis of one’s own moral principles), Honesty (valuing sincerity and the communication of the truth), Hedonism (importance given to achieving one’s own pleasure above other goals) and Social Recognition (importance given to being recognised and admired socially). The three second-order factors are: Social Values (Pro-Sociality, Social Commitment and Justice and Equality), Personal Values (Responsibility, Integrity and Honesty), and Individualistic Values (Hedonism and Social Recognition).

The instrument used is a seven-point scale (1: Not at all important; 2: Not very important; 3: Somewhat important; 4: Important; 5: Quite important; 6: Very important; 7: Most important).

The results of the original scale showed the good reliability of all dimensions (Social Values α = 0.89; Personal Values α = 0.89; Individualistic Values α = 0.80; Pro-Sociality α = 0.90; Social Commitment α = 0.90; Justice and Equality α = 0.86; Integrity α = 0.84; Responsibility α = 0.87; Honesty α = 0.87; Hedonism α = 0.84; Social Recognition α = 0.89).

### 2.3. Procedure

The test adaptation guidelines of the International Test Commission (ITC) [45] were followed for this study.

The first step, with the aim of obtaining evidence of content validity, was based on an analysis of the questionnaire and its justification by six university professors, from public and private universities in Spain, all of them with PhD degrees. The six experts were contacted because of their significant experience in the fields of education and psychology. Three of the experts were sport psychologists, one was a specialist in sport pedagogy and two were specialists in developmental psychology. After analysing the items and their suitability in terms of wording, and the congruence of each item in each dimension, the terminological adaptation was carried out to adapt them to the sporting context. After discussion and their respective comments, the experts reached consensus on the need for the modification of several items. Item 5, “Belonging to or participating in social organisations” from the Social Commitment factor, was replaced by “Belonging and participating in my team”. Item 6, “Being actively involved in groups, associations or organisations to which I belong” from the same factor, was adapted to “Actively involved in the sports club to which I belong”. Item 11, “Fight against social injustices” from the Justice and Equality factor, was replaced by “Fight against injustices in sport”. Finally, item 12, “Participate in a socially committed group” from Social Commitment, was reworded as “Engage socially with my team”. Furthermore, the heading of the instrument “Please rate on a scale of 1 to 7 how important the following issues are to you” was replaced by “Please rate on a scale of 1 to 7 how important the following issues are to you in sport”.

Once the final draft was completed, the questionnaire was administered to 10 athletes. In order to find out the possible differences in reading and comprehension by age, six boys and four girls were selected from the competition categories established in Spain: three from the Under 12 category (11–12 years), two from the Under 14 category (13–14 years), two from the Under 16 category (15–16 years) and three from the Under-19 category (17–19 years). They read the questionnaire, and all of them confirmed a correct understanding of the items. The final questionnaire is listed in Appendix A.

Subsequently, contact was made with different sports institutions with which the members of the research team collaborated. The objectives of the study were explained, and their participation was requested. The interested clubs and institutions facilitated contact with the sports coaches, also explaining the research approaches. The coaches provided the athletes with an informed consent form, which they had to give to their parents and return it signed, though only in the case of underage athletes.

The aims of the study, the relevance of their participation, and the confidential treatment of the data obtained were explained to the participants. The questionnaires were administered to each team of athletes before the training sessions and answered individually. Participation was voluntary. The researchers were present during the application of the tests in order to supervise the correct completion of the data and to resolve any doubts that might arise for the players.

### 2.4. Data Analysis

A screening of the data was carried out to verify the consistency of the responses and the absence of outliers in the variables analysed. IBM SPSS 21 was then used to calculate descriptive statistics.

Given the existence of previous research on the factor structure in the original version [40], a confirmatory factor analysis (CFA) was carried out using Amos Graphics 21 (IBM Statistics) in order to determine the instrument’s fit and reliability.

To assess the fit of the measurement model, several fit and error indices were calculated. These included: Chi-square (*χ*^2^)/degrees of freedom; the goodness-of-fit index (GFI); the adjusted goodness-of-fit index (AGFI); the comparative fit index (CFI); the root mean square error of approximation (RMSEA); standardised root mean square residual (SRMR).

In addition, an analysis of invariance was performed across three nested models to verify the equivalence of the model across different groups. Invariance was assessed using differences in *χ*^2^ tests, following the criteria of Cheung and Rensvold [46], where differences greater than 0.01 in CFI values indicated a lack of invariance. An analysis was conducted for gender, sport and age. The age groups were based on the WHO recommendations for disaggregation by age groups (10–14, 15–19 years) for the measurement of adolescents on different indicators [2]. By sport, an analysis was made between soccer, the sport with the highest number of players and participants and the rest of the sports.

Finally, it was decided to calculate the composite reliability index to assess reliability, as this analysis considers the presence of multidimensionality, unlike Cronbach’s alpha [47]. In terms of interpretation, index values above 0.7 in descriptive contexts or 0.9 in selective tests are considered acceptable [48].

## 3. Results

### 3.1. Descriptive Statistics

First, the data in all dimensions of the instrument did not follow a normal distribution according to the normality tests used (*p <* 0.05). This suggests that it is important to take into account the non-normality of the data when performing further statistical analyses and to consider the use of non-parametric methods or robust statistical techniques that do not rely on the assumption of normality.

Descriptive statistics for each item are shown in Table 1. The item means ranged between 4.19 (*SD* = 2.00; item 3) and 6.21 (*SD* = 1.02; item 17). Regarding the dimensions and their descriptions (Table 2), the highest mean was found in Honesty (*M* = 61.15; *SD* = 0.87), while the lowest average was for Social Recognition (*M* = 4.45; *SD* = 1.68). Moreover, it was observed that the mean of the highest level was in Personal Values (*M* = 5.89; *SD* = 0.75). Regarding the sample distribution, most of the variables showed a tendency towards negative skewness, suggesting that the data tended to accumulate towards the right end of the range. Furthermore, some variables showed a positive kurtosis, indicating a higher frequency of extreme values. In terms of distribution, the highest positive skewness was found in item 3 (0.003) and the highest negative skewness was found in item 21 (−1.74). Regarding the kurtosis indices, item 3 presented the highest negative value (−1.27), while the highest positive value was observed for item 21 (3.59).

In addition, the percentile rank of the Values Scale for Positive Youth Development in Sport is listed in Appendix B.

### 3.2. Confirmatory Factor Analysis (CFA)

A CFA was performed based on the factor structure defined by Antolin et al. [40]. This specific model contained 300 different sample moments, 73 parameters to estimate and 227 degrees of freedom. The method used to estimate the parameters was maximum likelihood (ML) with bootstrap due to a non-normal multivariate distribution (Mardia coefficient = 167.70, RC = 56.7).

The overall model fit was as follows: *χ*^2^ = 649.95 (*p* < 0.001); *χ*^2^/df = 2.86; goodness-of-fit index (GFI) = 0.91; adjusted goodness-of-fit index (AGFI) = 0.90; comparative fit index (CFI) = 0.90; root mean square error of approximation (RMSEA) = 0.056 (confidence interval of 90%, 0.051–0.061); standardised root mean square residual (SMSR) = 0.049.

The factor loadings were statistically significant (Table 3), with values ranging from 0.45 (item 1, Responsibility) to 0.94 (item 3, Social Recognition). Furthermore, it should be noted that significant and positive correlations were observed between the dimensions analysed (Table 4), which confirmed that they were related to each other. The highest observed relationship was between Pro-Sociality and Social Commitment (*r* = 0.577), and the lowest was between Social Recognition and Honesty (*r* = 0.114), although it remained significant. Finally, the correlation between the three value groups was also tested, and all were also significantly positive. The strongest relationship was observed between Social and Personal Values (*r* = 0.682, *p* < 0.001); the correlation between Social and Individualistic Values was moderate in magnitude (*r* = 0.459, *p* < 0.001) and between Personal and Individualistic Values (*r* = 0.366, *p* < 0.001).

### 3.3. Reliability Analysis

In addition to the correlations between factors, Table 4 shows the results obtained with respect to composite reliability. For this model, the highest reliability index was found for Social Recognition (0.89) and the lowest index for Integrity (0.72), at the limit of the values corresponding to good internal consistency.

### 3.4. Invariance Analysis

An invariance analysis was conducted to verify that the overall fit of the model was applicable independent of gender, age and sport. The analysis had the following structure: Model 1 (configuration model) is a base model without restrictions on the estimation of parameters in the different groups on which the subsequent comparisons were made. In this type of model, the indicators defining the measurement structure have the same configuration across the selected groups. Model 2 specified, in addition to the factor structure, the equality or invariance of the factor loadings between groups, and Model 3 added the correlations and variances of the factors.

With regards to gender (Table 5), the sample was divided between males (*n* = 392) and females (*n* = 207). The differences in CFI values were less than 0.01 for both Model 2 (∆CFI = −0.002) and Model 3 (∆CFI = −0.001) compared to Model 1. Therefore, factorial invariance between the genders of the athletes was established. Table 5 shows the obtained indices for invariance.

Regarding age (Table 6), two groups were obtained from the ranges of 10 to 14 years (n = 392) and 15 to 19 years (n = 207). Differences in the values of CFI were lower than 0.01 for both Model 2 (∆CFI = −0.002) and Model 3 (∆CFI = −0.002) compared to Model 1.

Finally, in terms of sport modality (Table 7), those who competed in soccer (n = 362) and those who competed in other sports (n = 237) were divided. It was also observed that differences in the values of the Comparative Fit Index (CFI) were less than 0.01 for both Model 2 (∆CFI = −0.001) and Model 3 (∆CFI = −0.001) compared to Model 1. Therefore, it was concluded that there was factorial invariance between the different groups for gender, age and sport modality.

## 4. Discussion

The positive youth development (PYD) approach has become increasingly relevant in recent times, with sport being a field of particular interest for the analysis of young people’s inner strengths and the development of positive sporting experiences. Based on the premise that PYD can be a suitable perspective from which to promote values in young people through sport, appropriate and valid instruments are needed for its assessment. Therefore, this study aimed to adapt and analyse the measurement properties of the Values Scale for Positive Youth Development [40] for its use in the sport context.

The results showed that the scale had adequate measurement properties. Firstly, in terms of construct validity, a good model fit was obtained, with a value of *χ*^2^/df = 2.86, which was between one and three, a criterion considered acceptable [49]. The different indices analysed also provide adequate data, since the values of the comparative fit index (CFI), goodness-of-fit index (GFI) and adjusted goodness-of-fit index (AGFI) have values equal to or greater than 0.90, and the error indices RMSEA and SMSR are below 0.80, all criteria proposed as satisfactory by different authors [50,51,52,53]. As for CFA, factor loadings above 0.50 were obtained for all items [52,54].

As for reliability, a composite reliability analysis was carried out as it takes into account the presence of multidimensionality [47]. The values ranged between 0.72 and 0.89, being acceptable when higher than 0.7 [48]. As previously noted, the original study by Antolín et al. [40] obtained values between 0.84 and 0.90, although using Cronbach’s alpha, which does not take into account the multidimensionality mentioned above [48].

An important aspect of the present study was the analysis of invariance, something that was not analysed in the original study [40]. The results confirm this factorial invariance between the gender, age and sport of athletes.

Therefore, all the results found were in accordance with those confirmed in the original questionnaire. The Antolin et al. [40] scale has been used in different research studies and contexts, mainly educational, showing good levels of validity and reliability. For example, the instrument has been used in studies on the prevention of bullying at school [55], and to analyse the relationship and influence between values and the criminalisation of misdemeanours and crimes [56]. It has also been adapted for use with Argentinian [57], Chilean [58] and Peruvian [59] adolescents, and has served as a basis for the development and validation of other scales [60,61]. In sport, we found only one study in which the instrument was used with athletes, specifically with young soccer players [14]. That study found that Personal Values are related to task orientation and Individualistic Values to ego orientation. Furthermore, the values of Responsibility, Integrity and Honesty predicted task orientation, while Social Recognition and Hedonism predicted ego orientation. Honesty and Responsibility were the main predictors of both task and social cohesion.

The scales assessed through the Values Scale for Positive Youth Development in Sport can be used for the analysis and understanding of important values in young athletes. Social values are related to both good social integration and a positive contribution to the community, which is emphasised in PYD models [21,62], and indicate an empathic and prosocial attitude in the adolescent, as well as an interest in collaborating in activities to help others [41]. Prosocial values are fundamental in sport. Behaviours such as encouraging, congratulating, giving positive feedback or constructive comments to teammates, as well as behaviour towards opponents, such as helping an injured opponent or asking to stop play when an opponent is injured, are examples of positive moral behaviour in sport [63], which should certainly be promoted.

Personal values imply a high level of personal security and strength to act consistently and follow one’s principles [40,41], and therefore, higher scores on this factor will show higher levels of responsibility, integrity and honesty, following their own convictions and moral principles [41]. In this respect, it is expected that they will be more integrated with their group, they will be more cooperative with their teammates to achieve the team’s objectives and they will seek greater companionship with their teammates [14]. In addition, previous studies have confirmed the relationship between personal values, attitudes and social behaviours [64,65,66].

Finally, individual values, without representing contra-values, have a less positive meaning than those identified previously [41]. While the values of hedonism and social recognition have been associated with ego orientation [14], which has been associated with aggression and unsportsmanlike or antisocial behaviour in sport, it has been suggested that the realm of sport is different in terms of moral exchanges with respect to other aspects of everyday life, where responsibilities are different, and reasoning within a game may lead participants to adopt greater egocentrism and a greater emphasis on victory [67]. We consider that hedonism, or the pursuit of pleasure or opportunities to have fun [41], in this age group should not be understood as problematic in the field of sport, as young athletes spend a lot of training and pursue enjoyment through the sport they like.

### Limitations and Future Research

However, despite the positive results of the study, we must also note several limitations. Firstly, the process of adaptation and validation of the Values Scale for Positive Adolescent Development in Sport was limited to its administration to Spanish athletes. Spain has its own culture and educational setting, so its use is conditioned to this specific context. Its application was also limited to the study ages covered by the instrument, adolescents between the ages of 11 and 19. Therefore, future research should analyse the properties of the instrument with athletes from different linguistic and cultural contexts. In addition, we must take into account the use of convenience sampling since the participants were selected in a non-random way. Furthermore, the impossibility of carrying out a retest prevented the verification of the constancy of response scores and thus the reliability of stability over time.

Regarding future research lines and practical applications, together with the aforementioned adaptation and validation of the instrument in other countries and languages, we believe that it would be interesting to analyse the values in samples of athletes from individual disciplines, since the participants in this study were all team modalities. Furthermore, the scale presented can be a useful tool to evaluate values in sport in general and also in sports programmes for education in values [68]. Of particular interest in this area would be the study of additional variables in relation to values and longitudinal and cross-cultural studies [69].

Furthermore, it is necessary to develop programmes based on sport from the perspective of the PYD, since the experiences carried out have had very positive results in different areas, with positive repercussions on physical, psychological and social levels, and especially for young people who are the most socially vulnerable [70,71,72,73]. Previous studies have confirmed the important characteristics of youth sports programmes that facilitate prosocial identities [74].

Finally, we must encourage clubs, sports institutions and coaches to opt for educational models within sport, as opposed to models where only competition is valued, and to make an effort to promote healthy learning environments that encourage young people to internalise positive values, develop adequately in society and help contribute to its improvement [14,15].

## 5. Conclusions

The Values Scale for Positive Youth Development in Sport is a valid and reliable instrument with adequate psychometric properties that make it an appropriate evaluation tool for use in sports contexts, starting from the positive youth development (PYD) approach.

Taking into account the limitations indicated, new studies are necessary that delve into its psychometric characteristics with different sports populations and several modalities. Its use should serve to understand the values that move young athletes in their practice and for coaches, clubs and sports institutions to become aware and work for the personal and moral development of their athletes.

## Figures and Tables

**Table 1:** Item descriptive statistics.

Item	Mean	SD	Skewness	Kurtosis
1	5.84	1.24	−1.19	1.23
2	4.75	1.90	−0.35	−1.08
3	4.19	2.00	0.00	−1.27
4	5.87	1.22	−1.10	0.93
5	5.08	1.58	−0.54	−0.47
6	5.38	1.44	−0.55	−0.40
7	5.55	1.35	−0.76	0.07
8	5.63	1.31	−0.78	0.08
9	6.07	1.15	−1.25	1.18
10	5.86	1.22	−1.13	1.01
11	5.62	1.45	−1.12	0.93
12	5.22	1.52	−0.75	0.11
13	6.05	1.17	−1.25	1.08
14	5.82	1.24	−0.96	0.54
15	5.77	1.54	−1.11	0.22
16	5.71	1.40	−1.15	1.05
17	6.21	1.02	−1.37	1.80
18	6.18	1.11	−1.57	2.48
19	4.41	1.98	−0.25	−1.12
20	5.90	1.48	−1.64	2.15
21	6.13	1.19	−1.74	3.59
22	5.54	1.39	−1.04	0.87
23	5.39	1.49	−0.89	0.20
24	5.30	1.48	−0.68	−0.13

**Table 2:** Descriptive statistics of the dimensions.

Dimension	Items	Mean	SD	Skewness	Kurtosis
Pro-Sociality	7, 8, 16	5.63	1.08	−0.81	0.58
Social Commitment	5, 6, 12	5.23	1.19	−0.46	−0.37
Justice and Equality	4, 10, 11	5.79	1.01	−0.88	0.38
Responsibility	1, 20, 21	5.96	0.97	−1.07	0.78
Integrity	14, 22, 24	5.56	0.96	−0.48	−0.08
Honesty	9, 17, 18	6.15	0.87	−1.16	1.36
Hedonism	13, 15, 23	5.74	1.06	−0.89	0.34
Social Recognition	2, 3, 19	4.45	1.68	−0.23	−0.91
Social Values	4, 5, 6, 7, 8, 10, 11, 12, 16	5.55	0.91	−0.50	−0.29
Personal Values	1, 9, 14, 17, 18, 20, 21, 22, 24	5.89	0.75	−1.00	1.48
Individualistic Values	2, 3, 13, 15, 19, 23	5.09	1.16	−0.25	−0.65

**Table 3:** Factor loadings.

Item	*λ*	*δ*	*R* ^2^
1	0.50	0.80	0.26
2	0.76	0.42	0.58
3	0.94	0.12	0.88
4	0.55	0.70	0.30
5	0.61	0.63	0.37
6	0.68	0.54	0.46
7	0.77	0.41	0.59
8	0.81	0.34	0.66
9	0.73	0.47	0.53
10	0.69	0.52	0.48
11	0.66	0.56	0.44
12	0.66	0.56	0.44
13	0.64	0.59	0.41
14	0.63	0.60	0.40
15	0.58	0.66	0.34
16	0.69	0.52	0.48
17	0.70	0.51	0.49
18	0.67	0.55	0.45
19	0.65	0.58	0.42
20	0.54	0.71	0.29
21	0.72	0.48	0.52
22	0.51	0.74	0.26
23	0.58	0.66	0.34
24	0.64	0.59	0.41

Note: *λ* = factor loadings; *δ* = error; *R*^2^ = variance.

**Table 4:** Inter-dimensional correlations and composite reliability.

Dimension	1	2	3	4	5	6	7	8
1. Pro-Sociality	**0.87**							
2. Social Commitment	0.577 **	**0.78**						
3. Justice and Equality	0.545 **	0.552 **	**0.76**					
4. Responsibility	0.451 **	0.437 **	0.431 **	**0.73**				
5. Integrity	0.488 **	0.487 **	0.415 **	0.382 **	**0.72**			
6. Honesty	0.518 **	0.464 **	0.534 **	0.522 **	0.458 **	**0.83**		
7. Hedonism	0.424 **	0.437 **	0.374 **	0.276 **	0.439 **	0.388 **	**0.73**	
8. Social Recognition	0.290 **	0.371 **	0.207 **	0.175 **	0.268 **	0.114 **	0.419 **	**0.89**

Note: ** The correlation is significant at level 0.01.

**Table 5:** Gender model invariance.

Model	*χ* ^2^	*gl*	*p*	NFI	CFI	RMSEA	RMSEA 90% CI	∆*χ*^2^	∆*gl*	∆CFI
1. Configuration model	903.74	454	<0.01	0.828	0.904	0.041	0.037–0.045	--	--	--
2. Invariant factor loadings	928.84	470	<0.01	0.823	0.902	0.040	0.037–0.044	25.1	16	0.002
3. Invariant factor correlations	938.99	475	<0.01	0.821	0.901	0.040	0.037–0.044	10.1	5	0.001

Note: *χ*^2^ = Chi-squared; *gl* = degrees of freedom; *p* = *p* value; NNFI = Bentler-Bonett Non-Normed Fit Index; CFI = Comparative Fit Index; RMSEA = Root Mean Square Error of Approximation; CI = confidence interval; ∆ = difference between values.

**Table 6:** Age model invariance.

Model	*χ* ^2^	*gl*	*p*	NFI	CFI	RMSEA	RMSEA 90% CI	∆*χ*^2^	∆*gl*	∆CFI
1. Configuration model	1223.30	448	<0.01	0.778	0.844	0.054	0.050–0.057	--	--	--
2. Invariant factor loadings	1248.66	464	<0.01	0.774	0.842	0.053	0.050–0.057	25.3	16	0.002
3. Invariant factor correlations	1265.29	469	<0.01	0.771	0.840	0.053	0.050–0.057	16.6	5	0.002

Note: *χ*^2^ = Chi-squared; *gl* = degrees of freedom; *p* = *p* value; NNFI = Bentler-Bonett Non-Normed Fit Index; CFI = Comparative Fit Index; RMSEA = Root Mean Square Error of Approximation; CI = confidence interval; ∆ = difference between values.

**Table 7:** Sports model invariance.

Model	*χ* ^2^	*gl*	*p*	NFI	CFI	RMSEA	RMSEA 90% CI	∆*χ*^2^	∆*gl*	∆CFI
1. Configuration model	850.74	448	<0.01	0.835	0.914	0.039	0.035–0.043	--	--	--
2. Invariant factor loadings	859.25	464	<0.01	0.833	0.913	0.038	0.034–0.042	8.5	16	0.001
3. Invariant factor correlations	868.56	469	<0.01	0.831	0.912	0.038	0.034–0.042	9.3	5	0.001

Note: *χ*^2^ = Chi-squared; *gl* = degrees of freedom; *p* = *p* value; NNFI = Bentler-Bonett Non-Normed Fit Index; CFI = Comparative Fit Index; RMSEA = Root Mean Square Error of Approximation; CI = confidence interval; ∆ = difference between values.

## Data Availability

Data may be obtained from the corresponding author.

## Appendix A

Escala de Valores para el Desarrollo Positivo Adolescente en el Deporte [Values Scale for Positive Youth Development in Sport]

Puntúa en una escala del 1 al 7 cómo son de importantes para ti las siguientes cuestiones en el deporte [Rate on a scale of 1 to 7 how important the following issues are to you in sport]:

**Nada Importante [Not Important]**	**Poco Importante [Not Very Important]**	**Algo Importante [Somewhat Important]**	**Importante [Important]**	**Bastante Importante [Quite Important]**	**Muy Importante [Very Important]**	**Lo más Importante [Most Important]**
1	2	3	4	5	6	7

1	Hacer las cosas lo mejor que se pueda, incluso cuando se tenga que hacer algo que no gusta [Doing things to the best of your ability, even when you have to do something you don’t like to do]	1	2	3	4	5	6	7
2	Recibir elogios de las demás personas [Receiving praise from others]	1	2	3	4	5	6	7
3	Ser admirado por los demás [To be admired by others]	1	2	3	4	5	6	7
4	Defender los derechos de los demás [Defending the rights of others]	1	2	3	4	5	6	7
5	Pertenecer y participar en mi equipo [Belonging and participating in my team]	1	2	3	4	5	6	7
6	Involucrarse de manera activa en el club deportivo al que pertenezco [Actively involved in the sports club to which I belong]	1	2	3	4	5	6	7
7	Dedicar parte del tiempo de uno a ayudar a los demás [Dedicate part of one’s time to helping others]	1	2	3	4	5	6	7
8	Responder a las necesidades de los demás [Responding to the needs of others]	1	2	3	4	5	6	7
9	Ser sincero con los demás [Being honest with others]	1	2	3	4	5	6	7
10	Ayudar a asegurar un trato justo para todo el mundo [Helping to ensure a fair deal for everyone]	1	2	3	4	5	6	7
11	Luchar contra las injusticias en el deporte [Fighting against injustice in sport]	1	2	3	4	5	6	7
12	Comprometerme socialmente con mi equipo [Engage socially with my team]	1	2	3	4	5	6	7
13	Buscar cualquier oportunidad para divertirse [Look for any opportunity to have fun]	1	2	3	4	5	6	7
14	Comportarse de acuerdo con los principios en los que se cree [Behave in accordance with the principles in which you believe]	1	2	3	4	5	6	7
15	Divertirse a toda costa [Having fun at all costs]	1	2	3	4	5	6	7
16	Trabajar para el bienestar de los demás [Working for the well-being of others]	1	2	3	4	5	6	7
17	Ser leal y fiel con los demás [Be loyal and faithful to others]	1	2	3	4	5	6	7
18	Ganarse la confianza de la gente siendo leal y honesto [Earning people’s trust by being loyal and honest]	1	2	3	4	5	6	7
19	Que las demás personas me admiren [To be admired by other people]	1	2	3	4	5	6	7
20	No culpar a otros de nuestros errores [Not blaming others for our mistakes]	1	2	3	4	5	6	7
21	Reconocer y asumir la responsabilidad cuando se ha hecho algo mal [Recognising and taking responsibility when something has been done wrong]	1	2	3	4	5	6	7
22	Defender lo que se cree aunque no sea bien visto por los demás [Stand up for what you believe in even if it is not well regarded by others]	1	2	3	4	5	6	7
23	Hacer cosas que resulten placenteras para uno mismo [Doing things that are pleasurable for oneself]	1	2	3	4	5	6	7
24	Actuar de acuerdo con lo que se piensa aunque no sea compartido por otros [Acting in accordance with what you think even if it is not shared by others]	1	2	3	4	5	6	7

## Appendix B

Percentile rank of the Values Scale for Positive Youth Development in Sport

**Pc**	**PS**	**SC**	**JE**	**R**	**I**	**Ho**	**He**	**SR**	**SV**	**PS**	**IV**
1	7	6	9	9	10	10	8	3	28	33	14
5	11	9	11	12	12	13	11	5	34	41	19
15	14	12	14	15	13	16	14	7	41	46	23
25	15	13	15	16	15	17	15	9	44	49	25
50	17	16	18	19	17	19	18	14	51	54	31
75	20	18	20	20	19	21	20	17	56	58	36
85	21	20	21	21	20	---	21	19	59	60	39
95	---	21	---	---	21	---	---	21	62	62	42
99	---	---	---	---	---	---	---	---	63	63	---
Mean	16.90	15.70	17.37	18.50	16.70	18.46	17.22	13.76	50.10	53.13	30.60
SD	3.25	3.56	3.03	2.91	2.88	2.62	3.19	5.06	8.22	6.81	7.00
Note: PS = Pro-Sociality; SC = Social Commitment; JE = Justice and Equality; R = Responsibility; I = Integrity; Ho = Honesty; He = Hedonism; SR = Social Recognition; SV = Social Values; PS = Personal Values; IV = Individualistic Values.											

## References

[1] WHO (2019). The Global Strategy for Women’s, Children’s, and Adolescents’ Health (2016–30). https://apps.who.int/gb/ebwha/pdf_files/WHA72/A72_30-en.pdf.

[2] WHO (2024). The Adolescent Health Indicators Recommended by the Global Action for Measurement of Adolescent Health: Guidance for Monitoring Adolescent Health at Country, Regional and Global Levels. https://iris.who.int/bitstream/handle/10665/376852/9789240092198-eng.pdf?sequence=1.

[3] Kroger J., Martinussen M., Marcia J.E. (2010). Identity status change during adolescence and young adulthood: A meta-analysis. J. Adolesc..

[4] Kromerova E., Šukys S. (2016). Adolescent involvement in sports activities and internalisation of moral values. Balt. J. Sport Health.

[5] Ahn J.S., Busque-Carrier M., Cho S., Rivard G. (2022). Value change across adolescent years: How do adolescents’ intrinsic and extrinsic values develop?. J. Res. Pers..

[6] Benish-Weisman M., Daniel E., Sneddon J., Lee J. (2019). The relations between values and prosocial behavior among children: The moderating role of age. Pers. Indiv. Differ..

[7] Cieciuch J., Davidov E., Algesheimer R. (2016). The stability and change of value structure and priorities in childhood: A longitudinal study. Soc. Dev..

[8] Lewis-Smith I., Pass L., Reynolds S. (2021). How adolescents understand their values: A qualitative study. Clin. Child Psychol. Psychiatry.

[9] León E., Boix S., Serrano M.A., Berengüí R., López-Walle J.M. (2018). Physical activity, exercise and health. Introduction to Sport Psychology.

[10] Pengpid S., Peltzer K. (2019). Sedentary behaviour, physical activity and life satisfaction, happiness and perceived health status in University students from 24 Countries. Int. J. Environ. Res. Public Health.

[11] Laborde S., Guillén F., Mosley E. (2016). Positive personality-trait-like individual differences in athletes from individual-and team sports and in non-athletes. Psychol. Sport Exerc..

[12] Wright P.M. (2023). Sport and Physical Activity for Positive Youth Development Related to Social and Emotional Learning: Reflections From the Know-Do Gap. Kinesiol. Rev..

[13] Gould D. (2019). The current youth sport landscape: Identifying critical research issues. Kinesiol. Rev..

[14] Berengüí R., Carralero R., Castejón M.A., Campos-Salinas J.A., Cantón E. (2022). Values, motivational orientation and team cohesion amongst youth soccer players. Int. J. Sports Sci. Coach..

[15] Berengüí R., Garcés de Los Fayos E.J. (2007). Valores en el deporte escolar: Estudio con profesores de educación física. Cuad. Psicol. Deporte.

[16] Light R., Harvey S. (2019). Positive Pedagogy for Sport Coaching.

[17] Purnomo S.A., Ma’mun A., Kusmaedi N., Hendrayana Y., Jermaina N., Amirudin A., Fitryona N., Sari D.M. (2024). Integration of Social Values Through Sport. Retos.

[18] Perkins D.F., Noam G.G. (2007). Characteristics of sports-based youth development programs. New Dir. Youth Dev..

[19] Lerner R.M., Almerigi J.B., Theokas C., Lerner J.V. (2005). Positive Youth Development A View of the Issues. J. Early Adolesc..

[20] Holt N.L., Neely K.C., Slater L.G., Camiré M., Côté J., Fraser-Thomas J., MacDonald D., Strachan L., Tamminen K.A. (2017). A grounded theory of positive youth development through sport based on results from a qualitative meta-study. Int. Rev. Sport Exerc. Psychol..

[21] Lerner R.M., Tirrell J.M., Dowling E.M., Geldhof G.J., Gestsdóttir S., Lerner J.V., King P.E., Williams K., Iraheta G., Sim A.T. (2019). The End of the Beginning: Evidence and Absences Studying Positive Youth Development in a Global Context. Adolesc. Res. Rev..

[22] Shek D., Dou D., Zhu X., Chai W. (2019). Positive youth development: Current perspectives. Adolesc. Health Med. Ther..

[23] Ross K.M., Tolan P.H., Dimitrova R., Wiium N. (2021). Positive youth development in the digital age: Expanding PYD to include digital settings. Handbook of Positive Youth Development: Advancing Research, Policy, and Practice in Global Contexts.

[24] Travers A.S., Mahalik J.R. (2019). Positive youth development as a protective factor for adolescents at risk for depression and alcohol use. Appl. Dev. Sci..

[25] Catalano R.F., Skinner M.L., Alvarado G., Kapungu C., Reavley N., Patton G.C., Jessee C., Plaut D., Moss C., Bennett K. (2019). Positive Youth Development Programs in Low- and Middle-Income Countries: A Conceptual Framework and Systematic Review of Efficacy. J. Adolesc. Health.

[26] Alvarado G., Skinner M., Plaut D., Moss C., Kapungu C., Reavley N. (2017). A Systematic Review of Positive Youth Development Programs in Low-and Middle-Income Countries.

[27] Holt N.L., Sehn Z.L., Spence J.C., Newton A.S., Ball G.D. (2012). Physical education and sport programs at an inner city school: Exploring possibilities for positive youth development. Phys. Educ. Sport Pedagog..

[28] Smith K.L., Jackson D., Weir P.L. (2024). Relative Age and Positive Youth Development in Youth Sport: Do Developmental Assets Play a Role in Creating Advantage Reversals in Female Soccer?. Sports.

[29] Barcza-Renner K., Anderson-Butcher D., Amorose A.J., Wade-Mdivanian R. (2022). Examining the Impact of Counselor and Peer Support on Social Skill Development in a Sport-Based Positive Youth Development Program. J. Sport Behav..

[30] Anderson-Butcher D. (2019). Youth sport as a vehicle for social development. Kinesiol. Rev..

[31] Bean C., Forneris T. (2017). Is Life Skill Development a By Product of Sport Participation? Perceptions of Youth Sport Coaches. J. Appl. Sport Psychol..

[32] Rokeach M. (1973). The Nature of Human Values.

[33] Schwartz S.H. (1992). Universals in the content and structure of values: Theoretical advances and empirical tests in 20 countries. Adv. Exp. Soc. Psychol..

[34] Schwartz S.H., Cieciuch J., Vecchione M., Davidov E., Fischer R., Beierlein C., Ramos A., Verkasalo M., Lönnqvist J.E., Demirutku K. (2012). Refining the theory of basic individual values. J. Pers. Soc. Psychol..

[35] Gordon L.V. (2003). Cuestionario de Valores Personales (SPV).

[36] Lee M.J., Whitehead J., Balchin N. (2000). The measurement of values in youth sport: Development of youth sport values questionnaire. J. Sport Exerc. Psychol..

[37] Lee M.J., Whitehead J., Ntoumanis N., Hatzigeorgiadis A. (2008). Relationships among values, achievement orientations, and attitudes in youth sport. J. Sport Exerc. Psychol..

[38] Mortimer H., Whitehead J., Kavussanu M., Gürpınar B., Ring C. (2021). Values and clean sport. J. Sports Sci..

[39] Pena-Pérez X., Portela I., González-Silva J., Fuentes-García J.P., Martínez-Patiño M.J. (2023). Design and validation of a questionnaire in Spanish on sports and Olympic values. Retos.

[40] Antolín L., Oliva A., Pertegal M.A., López A.M. (2011). Development and validation of a scale to assess positive youth development values. Psicothema.

[41] Oliva A., Reina M.C., Hernando A., Antolín L., Pertegal M.A., Parra A., Ríos M., Estévez R.M., Pascual D.M. (2011). Assets for Positive Development and Mental Health in Adolescence.

[42] Hartman R.S. (1973). The Hartman Value Profile (HVP).

[43] Sanz de Acebo M.L., Ugarte M., Lumbreras M.V. (2003). Desarrollo y validación de un cuestionario de metas para adolescentes. Psicothema.

[44] De la Fuente J., Peralta F.J., Sánchez M.D. (2006). Valores sociopersonales y problemas de convivencia en la Educación Secundaria. Electron. J. Res. Educ. Psychol..

[45] Hernández A., Hidalgo M.D., Hambleton R.K., Gómez-Benito J. (2020). International Test Commission guidelines for test adaptation: A criterion checklist. Psicothema.

[46] Cheung G.W., Rensvold R.B. (2002). Evaluating goodness-of-fit indexes for testing measurement invariance. Struct. Equ. Model..

[47] Dunn T.J., Baguley T., Brunsden V. (2014). From alpha to omega: A practical solution to the pervasive problem of internal consistency estimation. Br. J. Psychol..

[48] Prieto G., Delgado A.R. (2010). Reliability and validity. Papeles Psicólogo.

[49] Jöreskog K.G. (1970). A general method for analysis of covariance structures. Biometrika.

[50] Arias B., Verdugo M.A., Crespo M., Badía M., Arias B. (2008). Development of an example of a confirmatory factor analysis with LISREL, AMOS and SAS. Metodología en La Investigación Sobre Discapacidad. Introducción al Uso de Las Ecuaciones Estructurales.

[51] Browne M.W., Cudeck R., Bollen K.A., Long J.S. (1993). Alternative ways of assessing model fit. Testing Structural Equation Models.

[52] Hu L.T., Bentler P.M. (1999). Cutoff criteria for fit indexes in covariance structure analysis: Conventional criteria versus new alternatives. Struct. Equ. Model..

[53] Schreiber J.B., Nora A., Stage F.K., Barlow E.A., King J. (2006). Reporting structural equation modeling and confirmatory factor analysis results: A review. J. Educ. Res..

[54] Wheaton B., Muthen B., Alwin D.F., Summers G. (1977). Assessing reliability and stability in panel models. Sociol. Methodol..

[55] Aguilar A.S., Andreu J.M., Peña M.E. (2014). La Prevención Del Maltrato en La Escuela, Experiencia de Un Programa Entre Alumnos de Educación Media.

[56] Rodríguez L.M., Schönfeld F., Hess C., Moreno J., Benitez M. (2017). Valores y Penalización de Actos. Un Estudio Para el Desarrollo Positivo Adolescente.

[57] Rodriguez L.M., Hess C., Schönfeld F., Mesurado B. (2023). Adaptación a población argentina de la Escala de Valores para el Desarrollo Positivo Adolescente. Rev. CES Psicol..

[58] Gálvez-Nieto J.L., Vera D., Trizano I., Polanco K., Salvo S. (2018). Psychometric Properties of the Reduced Version of the Positive Adolescent Development Value Scale (EVDPA-R) in Chilean Students. Rev. Colomb. Psicol..

[59] Campos Del Carpio D. (2019). Escala de Valores Para el Desarrollo Positivo Adolescente (EVDPA-R): Propiedades Psicométricas en una Muestra de Lima. Doctoral Thesis.

[60] Hernández-Lalinde J.D., García-Álvarez D., Soler M.J. (2021). FDA scale for the measurement of adolescent development factors and their prediction in psychological well-being. Retos.

[61] Sánchez-Oliva D., Sánchez P., Leo F., Amado D., García-Calvo T. (2013). Development and validation of a questionnaire to examine perception of positive behaviours in physical education classes. Cult. Educ..

[62] Lerner R.M. (2004). Liberty: Thriving and Civic Engagement among American Youth.

[63] Kavussanu M., Al-Yaaribi A. (2019). Prosocial and antisocial behaviour in sport. Int. J. Sport Exerc. Psychol..

[64] Boer D., Fischer R. (2013). How and when do personal values guide our attitudes and sociality? Explaining cross-cultural variability in attitude-value linkages. Psychol. Bull..

[65] Danioni F., Barni D. (2019). The relations between adolescents’ personal values and prosocial and antisocial behaviours in team sports. Int. J. Sport Exerc. Psychol..

[66] Adell F.L., Castillo I., Alvarez O. (2019). Personal and sport values, goal orientations, and moral attitudes in youth basketball. Rev. Psicol. Deporte.

[67] Shield D., Bredemeier B. (1995). Character Development and Physical Activity.

[68] Whitehead J., Telfer H., Lambert J. (2013). Values in Youth Sport and Physical Education.

[69] Olmos-Gómez M.C., Portillo-Sánchez R., Mohamed-Mohand L., Estrada-Vidal L.I. (2024). Promotion of Values Education (Factors Involved in Prosocial Behaviors and Volunteering). Eur. J. Investig. Health Psychol. Educ..

[70] Bruner M.W., McLaren C.D., Sutcliffe J.T., Gardner L.A., Lubans D.R., Smith J.J., Vella S.A. (2021). The effect of sport-based interventions on positive youth development: A systematic review and meta-analysis. Int. Rev. Sport Exerc. Psychol..

[71] Hermens N., Super S., Verkooijen K.T., Koelen M.A. (2017). A systematic review of life skill development through sports programs serving socially vulnerable youth. Res. Q. Exerc. Sport.

[72] Pozo P., Grao-Cruces A., Pérez-Ordás R. (2018). Teaching personal and social responsibility model-based programmes in physical education: A systematic review. Eur. Phys. Educ. Rev..

[73] Whitley M.A., Massey W.V., Camiré M., Boutet M., Borbee A. (2019). Sport-based youth development interventions in the United States: A systematic review. BMC Public Health.

[74] Lee W., Jones G.J., Wegner C. (2023). It’s all relative: Examining the influence of social identity on sport-based youth development. Sport Manag. Rev..

